# The Effect of Local and Landscape-Level Characteristics on the Abundance of Forest Birds in Early-Successional Habitats during the Post-Fledging Season in Western Massachusetts

**DOI:** 10.1371/journal.pone.0106398

**Published:** 2014-08-29

**Authors:** Michelle A. Labbe, David I. King

**Affiliations:** 1 Department of Environmental Conservation, University of Massachusetts Amherst, Amherst, Massachusetts, United States of America; 2 United States Forest Service Northern Research Station, University of Massachusetts Amherst, Amherst, Massachusetts, United States of America; University of South Carolina, United States of America

## Abstract

Many species of mature forest-nesting birds (“forest birds”) undergo a pronounced shift in habitat use during the post-fledging period and move from their forest nesting sites into areas of early-successional vegetation. Mortality is high during this period, thus understanding the resource requirements of post-fledging birds has implications for conservation. Efforts to identify predictors of abundance of forest birds in patches of early-successional habitats have so far been equivocal, yet these previous studies have primarily focused on contiguously forested landscapes and the potential for landscape-scale influences in more fragmented and modified landscapes is largely unknown. Landscape composition can have a strong influence on the abundance and productivity of forest birds during the nesting period, and could therefore affect the number of forest birds in the landscape available to colonize early-successional habitats during the post-fledging period. Therefore, the inclusion of landscape characteristics should increase the explanatory power of models of forest bird abundance in early-successional habitat patches during the post-fledging period. We examined forest bird abundance and body condition in relation to landscape and habitat characteristics of 15 early-successional sites during the post-fledging season in Massachusetts. The abundance of forest birds was influenced by within-patch habitat characteristics, however the explanatory power of these models was significantly increased by the inclusion of landscape fragmentation and the abundance of forest birds in adjacent forest during the nesting period for some species and age groups. Our findings show that including factors beyond the patch scale can explain additional variation in the abundance of forest birds in early-successional habitats during the post-fledging period. We conclude that landscape composition should be considered when siting early-successional habitat to maximize its benefit to forest birds during the post-fledging period, and should also be included in future investigations of post-fledging habitat use by forest birds.

## Introduction

To address long-term declines in populations of Neotropical migratory bird species, conservationists need to understand the resource requirements and factors affecting survival of these species throughout their lifecycle. Historically research has focused primarily on the nesting season and to a lesser extent, the migratory and wintering periods. However, recent reports documenting low juvenile survival during the post-fledging period [1]–[4] – the time period after the young fledge from the nest and before initiation of migration – have turned attention toward this poorly understood, yet critical phase of their lifecycle. Mortality can be high during this period, particularly for juveniles who have limited mobility and are inexperienced at foraging and evading predators [2], [5], [6]. Adults may also be more vulnerable during this time due to impaired flight capability while they undergo a pre-basic molt [10].

Previous studies have shown that mature forest-nesting birds (“forest birds”), a group including species of particular conservation concern [7], undergo a pronounced change in habitat use during the post-fledging season, and move from their forested nesting sites into areas of early-successional vegetation. This habitat shift has been attributed to birds seeking out areas with increased understory vegetation and food abundance, resources presumed to enhance survival [3], [8]–[11]. While past research has greatly improved our understanding of post-fledging ecology [3], [4], [9], [10], [12], [13], these findings vary considerably relative to the factors they cite as influencing the abundance of forest birds during the post-fledging period, some highlighting the importance of fruit abundance and others emphasizing dense vegetation providing protection for predators. One possible explanation for the apparent difficulty in identifying consistent patterns is the potential influence of landscape on the abundance of forest birds in early-successional habitats. Studies that have investigated this subject have focused on contiguously forested landscapes and the relative influence of landscape in more fragmented and modified landscapes is largely unknown. The composition of the surrounding landscape is known to have a strong influence on the abundance and productivity of forest birds during the nesting period [14]–[17], and could therefore affect the number of forest birds available to colonize early-successional habitats during the post-fledging period. Thus, there is reason to expect that the inclusion of landscape factors into models of forest bird abundance during the post-fledging period will increase our ability to explain variation in forest bird abundance in early-successional habitats during the post-fledging period.

The objective of this study was to examine whether the inclusion of landscape characteristics increase the explanatory power of models of forest bird abundance in early-successional habitats during the post-fledging period. Specifically, we studied how captures of forest birds in early-successional habitats in Massachusetts are related to (1) local habitat characteristics, including vegetation structure and composition, fruit abundance, and the prevalence of invasive plants, and (2) the composition and configuration of forest in the landscape surrounding habitat patches and the abundance of breeding birds in forests adjacent to habitat patches. Lastly, we examined the effects of local and landscape characteristics on the body condition of post-fledging birds.

## Materials and Methods

### Ethics Statement

The protocol for this study was approved by Institutional Animal Care and Use Committee (IACUC) of the University of Massachusetts (permit #26-02-05). Permits required for bird sampling were obtained prior to the initiation of this study (USGS Banding permit #23140 issued to the USFS Northern Research Station). No listed species were sampled during this study.

### Study area

**Figure 1:**
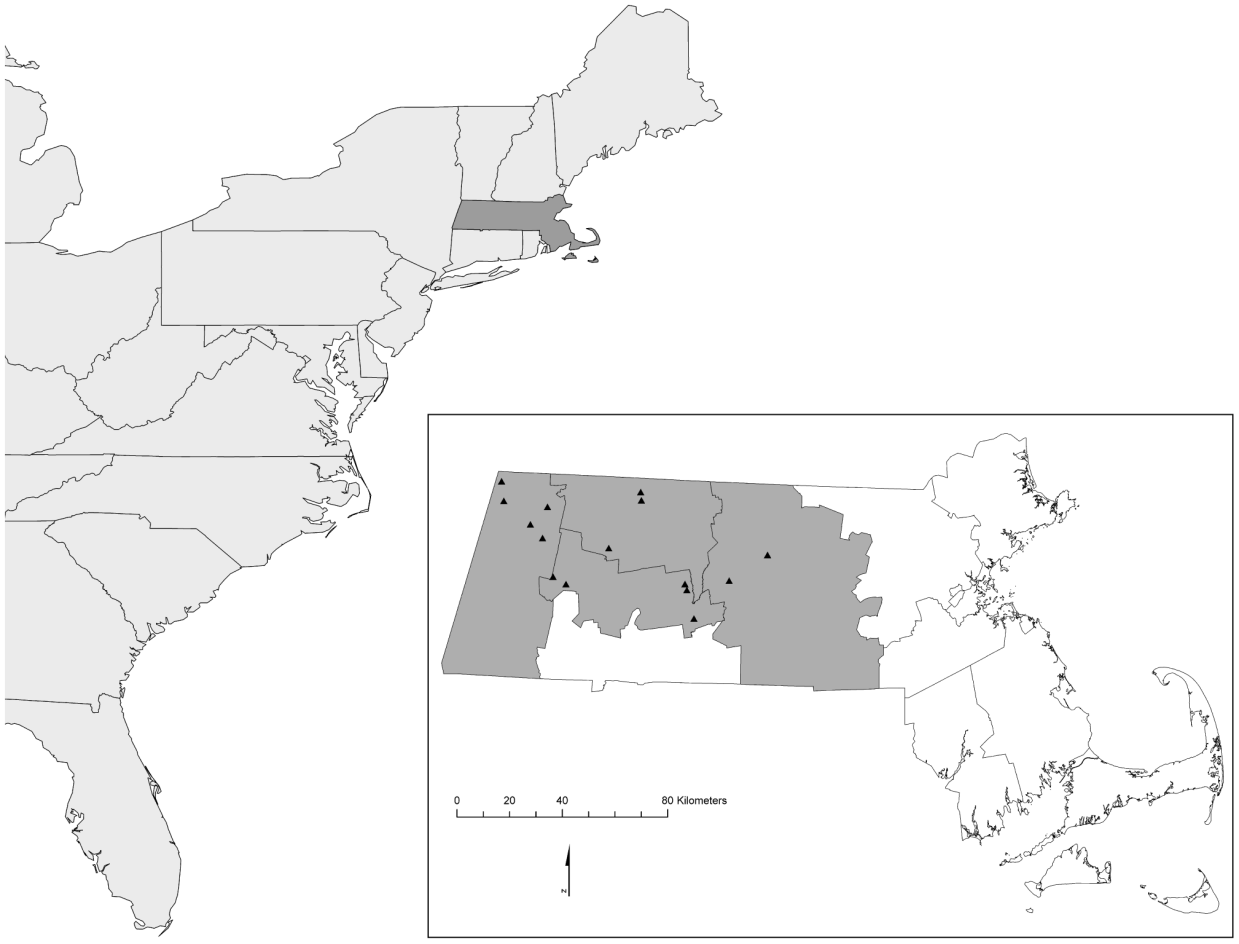
Study area and location of study sites in Berkshire, Franklin, Hampshire, and Worcester counties Massachusetts.

This study was conducted in 2006 and 2007 at 15 sites in Berkshire, Franklin, Hampshire, and Worcester counties, Massachusetts (Figure 1; see Appendix S1 for site information). Forests in the region are predominantly transitional and northern hardwoods dominated by maples (*Acer rubrum, A. saccharum*), birches (*Betula lenta, B. papyrifera, B. alleghaniensis*), red oak (*Quercus rubra*), eastern hemlock (*Tsuga canadensis*), white pine (*Pinus strobes*), American beech (*Fagus grandifolia*), and white ash (*Fraxinus americana*), with spruce (*Picea* spp.) and fir (*Abies* spp.) at higher elevations. Study sites included 11 state-owned wildlife openings and 4 regenerating clearcuts on private and public land. Wildlife openings were maintained by mechanical treatment approximately every 10 years and had been treated within 7 to 8 years prior to this study; regenerating clearcuts were between 6 and 7 years post-harvest. Sites ranged in size from 5 to 19 ha, were a minimum distance of 2.5 km apart, separated by mixed land use, and were characterized by shrubs, herbaceous plants and scattered trees – the height of which varied depending on management history and site characteristics. Common shrubs included native species in the genera *Cornus*, *Rubus*, *Rhus, Vaccinium*, and *Spirea*, as well as non-native species such as honeysuckle (*Lonicera* spp.), common buckthorn (*Rhamnus cathartica*), glossy buckthorn (*Frangula alnus*), autumn olive (*Elaeagnus umbellata*), and multiflora rose (*Rosa multiflora*). Common saplings included red maple, birches, white ash, black cherry (*Prunus serotina*), white pine, red oak, and pin cherry (*Prunus pensylvanica*) and residual trees were typically apple (*Malus* spp.), black cherry, and white ash. All early-successional sites were surrounded by mature forest. The sites sampled encompassed all of the suitable clearcuts and wildlife openings available in the study region and year, and thus we were unable to randomly select study sites. Sites that we considered unsuitable were those that had been recently treated (≤2 years) and had limited vegetation regrowth, or could not accommodate a 250×100 m grid of 10 mist nets due to the size and/or shape of the site.

### Bird sampling in early-successional habitats

Bird abundance was surveyed using standardized mist netting between mid-July and mid-August of each study year. Eight sites were surveyed in 2006 and ten sites were surveyed in 2007. Sites were visited twice within the study season, with approximately 14 days between visits. Ten mist nets (12 m long, 3 m high, 32 mm denier) were arranged in a 250×100 m grid with a distance of 50 m between nets and at least 25 m between each net and the edge of the habitat type. Nets were opened at sunrise and operated for 5 hours per visit (weather permitting). All birds captured, except Ruby-throated Hummingbirds (*Archilochus colubris*), were banded with a United States Geological Survey (USGS) aluminum band, and information on age, sex, mass, molt, fat, breeding condition (evidence of brood patch or cloacal protuberance) and unflattened wing chord were collected. In 2006, we took tarsus measurements for species anticipated to have sufficient captures in our sites for individual analyses and in 2007, we included additional species based on our 2006 capture rates. Age (classified as either juvenile or adult) was determined by degree of skull ossification, plumage, molt patterns, and evidence of cloacal protuberance or brood patch [18]. The post-fledging status of individual birds was indicated by the presence of molt and/or the absence of migratory fattening.

### Bird sampling in forest

In 2007, we expanded our sampling to include an additional landscape factor, the local abundance of nesting forest birds available to colonize sites during the post-fledging period. We quantified nesting bird abundance at 9 sites in 2007 at 5 points in the forest adjacent to each site using 10-minute, 50-m radius point counts [19]. Points were located using a random starting point 150 m from the patch edge (defined as the center of the outermost canopy tree [20], and at that same distance from the edge 250 m apart thereafter. Each point was surveyed 3 times between 0500–1000 hours on calm days with no precipitation in June and early July 2007.

### Quantifying land use characteristics

We used the 2005 MassGIS statewide land use layer based on 0.5 m resolution digital orthoimagery (see Acknowledgements) in ArcGIS 9.2 (ESRI) to quantify the percent cover of forest, agriculture, development, and areas dominated by early-successional vegetation within a 1-km radius of the center of each study plot. We chose the distance of 1 km because previous studies have shown that this corresponds to a spatial scale at which forest-nesting birds interact with their environment during the post-fledging period [21]–[24]. MassGIS categories representing developed or human-disturbed land use were grouped into the single category “developed,” and all agricultural categories were grouped as “agriculture.” Because we were interested in how the composition of nesting habitat in the landscape may affect post-fledging birds, we calculated the total number of patches and mean patch size for forest cover within the 1-km buffer. We used principal components analysis (PCA) to combine the configuration metrics and the percent land cover categories. The percent land cover categories were arcsine-square root transformed and the composition metrics were log-transformed prior to the PCA. We retained two PCA axes, which explained 89% of variation in the original data (Table 1). The first component (“fragmentation”) described a gradient with increasing percent cover of developed, agricultural, and early-successional land classes and total forest patches, and decreasing contiguous forest cover and mean forest patch size. The second component (“early-successional”) described a gradient of increasing early-successional land cover and decreasing agriculture land cover.

**Table 1:** Results from a principle component analysis (PCA) performed on land use classes and forest configuration metrics^a^.

	PC1 (“fragmentation”)	PC2 (“early-successional”)
Eigenvalue	4.3	1.03
% variance explained	0.72	0.17
Cumulative proportion	0.72	0.89
Component loadings:		
% Forest	−0.98	0.02
% Agriculture	0.67	−0.66
% Early-successional	0.67	0.69
% Developed	0.79	−0.3
# Forest patches	0.95	0.13
Mean patch (ha.)	−0.96	−0.12

a Variables were measured within a 1-km radius of each study site and included the percent cover of forest (%Forest), agriculture (%Agriculture), areas dominated by early-successional vegetation (%Early-successional) and human-disturbed and/or developed land (%Developed); and forest configuration metrics, total patches (# Forest patches) and mean patch size (Mean patch).

### Quantifying habitat characteristics

We used a point intercept method to measure vegetation characteristics and fruit abundance at each net within each site. Specifically, 10 random points were located within a 10-m radius area around each net using random compass bearings and choosing distances from the plot center with a random number sheet. At each of the ten points, we recorded the height and the species of the tallest vegetation. Fruit was counted within 1-m diameter circles centered on each of the 10 random points and categorized as unripe, ripe, or desiccated. Vegetation data was combined into life form categories that reflected vegetation type and structure. These categories have been shown to be useful descriptors of early-successional habitats [25] and included graminoids, ferns and forbs, low broadleaved shrubs (<2 m), broadleaved shrubs (2–5 m), and broadleaved trees (>5 m). Non-native plants were primarily tree and shrub species; thus, invasive cover was calculated as a percentage of total tree and shrub cover.

### Modeling nesting bird abundance in forest

Estimates of forest breeding bird abundance were calculated for forest species that comprised at least 5% of total forest bird captures and occurred at 30% or more of sites as determined by both point count and mist net data. We chose this cutoff criterion because it was the threshold at which a species had sufficient numbers to both compute its detection estimate and model its abundance. Estimates for individual species were corrected for heterogeneity of detection probabilities using N-mixture models [26]. N-mixture models estimate both the mean probability of detection and an adjusted mean number of birds per plot. Detections by sight, song and call were all included in the analysis. We calculated the mean breeding bird abundance for forest species combined to examine it in relation to the total captures of juvenile and adult forest nesting birds in early-successional sites during the post-fledging period.

### Modeling post-fledging bird abundance in early-successional habitats

Abundance models were constructed for forest bird species combined as well as separately for species comprising at least 5% of total forest bird captures and occurring at 30% or more of sites. We analyzed juvenile and adult birds separately because they may experience different ecological pressures due to differing mobility and experience during the post-fledging season [1]–[4], and therefore may respond differently to environmental variables. Only the captures of birds classified as mature forest-nesting species were included in analyses of abundance and condition; these classifications were based on species' primary habitat associations [27]–[29]. Although the breadth of habitat used by some of these species may differ by state or region, for this study we feel our classifications are appropriate, as they are consistent with studies previously conducted in our sites and other clearcuts and wildlife openings in the region [e.g. 30], and thus will allow for direct comparison between these studies. Paired t-tests were used to test whether capture rates of adults or juvenile forest-nesting species differed between survey dates for each site within a given year. Since there were no significant differences in capture rates between survey days, captures were combined over survey days for each study site for each year.

We examined the influence of local and landscape-level factors on the captures of post-fledging forest birds using generalized linear mixed models (GLMM) specified with a Poisson distribution with a log-link function and fitted with Laplace estimation. Nets were considered a sample unit and were nested within sites; therefore, we included “site” as a random effect. We included “effort” as an offset to account for the total hours that a net was operated and the covariate, “year” because data were collected in multiple years. In cases where year was not present in supported models (based on AIC_c_ [31]), and was found to have no significant effect when tested against the null model for all species and age classes, year was removed from those models. Explanatory variables of interest included vegetation life form categories, fruit abundance, proportion of non-native trees and shrubs, and landscape principal components (“fragmentation” and “early-successional”). Prior to analysis, distributions for all variables were examined using histograms and scatter plots, and where appropriate, explanatory variables were log-transformed, centered and scaled to unit variance to improve model interpretation [32]. Analyses were performed in R version 2.14 with the function lmer [33], [34].

We used an information criterion adjusted for small sample size (AIC_c_) [31] to compare and select candidate models constructed with additive combinations of habitat and landscape variables. Due to the large number of biologically plausible models representing our working hypothesis, we used a manual forward-selection process to construct candidate models based upon AIC_c_ values. Specifically, we modeled each variable independently and progressively built models with multiple variables by retaining those variables that lowered the AIC_c_ relative to the null model and had a partial effect significant at α = 0.05 as determined by goodness-of-fit tests [35]. We also included the null model in each candidate model set. Models were ranked by their AIC_c_ values [31]. Models with AIC_c_ values within two units of the best model were considered supported and model terms with 95% confidence intervals that did not include zero were considered strongly supported. Overdispersion was assessed for top models by comparing the estimated dispersion parameter to one. This process of model selection was carried out for adults and juveniles of forest species combined and the five focal species that met our criteria for separate analysis. To examine the relationship between breeding bird abundance and captures of post-fledging birds, we repeated the model selection process described above with estimates of forest breeding bird abundance for the subset of sites that had point count data (hereafter, “point count subset”). This subset included two years of habitat and capture data for the 4 sites that were mist-netted in 2006 and 2007 and one year of habitat and capture data for 5 sites that were mist-netted in 2007 only. Repeating the model selection process allowed us to examine the importance of breeding bird populations in the surrounding landscape relative to habitat and landscape characteristics.

The contribution of landscape variables and forest bird abundance in adjacent forest to models of mist net captures in early-successional habitats was further evaluated using likelihood ratio tests to compare top models with and without terms for these variables [36]. The outcomes of these tests were considered significant at the α = 0.05 level.

### Body condition

To complement our analysis of forest bird abundance within early-successional habitats, we examined the body condition indices of focal forest bird species using mass/wing chord as an index of body condition for focal species and age classes [37]. The relationships between condition and habitat and landscape variables were analyzed using univariate restricted maximum likelihood (REML) models of habitat and landscape variables with site as a random effect with the lmer function in R version 2.14 [33], [34]. Significance of fixed effects was determined using parametric bootstrap based tests (10,000 iterations); α = 0.05 was considered statically significant [38].

## Results

We captured 1,825 individuals of 60 species during 2006 and 2007. Of these captures, 478 (27%) individuals and 26 (43%) species were mature forest-nesting birds [27]–[29]. The remaining captures were birds that either nest in early-successional or grassland habitats, or were habitat generalists. We were able to age 95% of the forest birds we captured, and 51% percent were adults and the remainder were juveniles. Forest-nesting bird species that met our criteria for analysis were American Redstart, Black-capped Chickadee, Ovenbird, Red-eyed Vireo, and Veery (See Appendix S2 in supporting material for scientific names). These five species accounted for 71% of forest-bird mist net captures and 73% of all forest birds detected during nesting-season point counts surveys (total point count detections were 70, 77, 120, 98, and 100, respectively).

### Bird-habitat associations – patch-level

A single model was supported describing the relationship between adults of all species combined and patch-level habitat, and indicated that overall adult birds were negatively associated with cover of invasive plants and positively related to fruit abundance and tree cover (Table 2). However, habitat associations varied among species. Of the five abundances modeled for adults of individual species, two were improved by the inclusion of graminoid cover; this relationship was positive for Red-eyed Vireos and negative for Veeries. Two of the five abundances modeled were improved by the inclusion of invasives; this relationship was negative for both Red-eyed Vireos and Black-capped Chickadees. Two of five abundances modeled were improved by the inclusion of tall shrubs and this relationship was positive for both Veeries and for American Redstarts. The inclusion of trees, fruit, and ferns and forbs each improved one of five abundances modeled, and each of these variables were positively associated with Red-eyed Vireos.

**Table 2:** Model selection results and parameter estimates with standard errors for Poisson regression analysis of adult and juvenile forest bird captures in early-successional habitats.

			Patch-level							Landscape-level						
	N	Intcpt	fnfb	gram	lowsh	inv	tallsh	fruit	tree	PC1	PC2	year	AIC_c_	ΔAIC_c_	ω	R^2^
*Adults*																
ALSP	251	**−3.02(0.42)**				**−0.41(0.12)**		**0.28(0.1)**	**1.02(0.21)**			**0.87(0.24)**	345	0	0.99	0.53
REVI	102	**−5.8(0.84)**	**0.4(0.16)**	**0.36(0.14)**		**−0.82(0.21)**		**0.53(0.16)**	**2.02(0.37)**	**−0.89(0.42)**		**1.1(0.41)**	200.4	0	0.69	0.73
		**−5.95(0.98)**	**0.39(0.16)**	**0.36(0.14)**		**−0.84(0.21)**		**0.53(0.16)**	**2.03(0.38)**			**1.09(0.41)**	202.3	1.9	0.27	0.73
BCCH	36	**−5.14(0.45)**				**−0.54(0.25)**						**1.35(0.47)**	152.7	0	0.33	0.21
VEER	19	**−5.13(0.39)**					**0.52(0.26)**				**−1.03(0.44)**		98.7	0	0.44	0.08
		**−5.1(0.38)**		−0.57(0.29)							**−1.11(0.43)**		99	0.3	0.38	0.08
OVEN	13	**−5.93(0.60)**							**1.03(0.52)**				66.4	0	0.82	0.21
AMRE	26	**−4.46(0.25)**					**0.71(0.23)**			**−0.23(0.12)**			111	0	0.64	0.06
*Juveniles*																
ALSP	232	**−2.43(0.22)**		**−0.26(0.11)**			**0.3(0.09)**						279.9	0	0.41	0.46
		**−2.44(0.24)**		**−0.22(0.11)**			**0.29(0.09)**	0.15(0.11)					280	0.10	0.40	0.47
REVI	13	**−5.02(0.57)**						**1.59(0.61)**				**−2.21(0.83)**	65.6	0	0.98	0.18
BCCH	27	**−4.91(0.43)**				−0.53(0.33)							122.4	0	0.63	0.15
		**−4.8(0.42)**											123.5	1.1	0.37	0.14
VEER	53	**−3.93(0.23)**		**−0.55(0.22)**					**0.53(0.20)**	**−0.21(0.11)**			143.3	0	0.64	0.27
		**−3.98(0.26)**		**−0.54(0.22)**					**0.46(0.22)**				144.9	1.6	0.28	0.27
OVEN	25	**−4.84(0.42)**											110.9	0.9	0.39	0.2
AMRE	24	**−5.45(0.52)**					**0.89(0.30)**						87	0	0.99	0.57

Data were collected from 15 early-successional habitats during Jun.-Aug., 2006 and 2007 in Berkshire, Franklin, Hampshire, and Worcester counties, MA. Only models within 2 ΔAIC_c_ unit of top model are shown. Bold text indicates coefficients with 95% confidence intervals that do not include zero. Variables are as follows: ALSP = all species combined; REVI = Red-eyed Vireo; BCCH = Black-capped Chickadee; VEER = Veery; OVEN = Ovenbird; AMRE = American Redstart; Intcpt = Intercept; fnfb = ferns and forbs; gram = graminoids; lowsh = low shrubs (<2 m); inv = non-native trees and shrubs; tallsh = tall shrubs (>2 m); fruit = fruit abundance; tree = trees (>5 m); PC1 = "fragmentation"; PC2 = "early-successional"; year = year.

A single model was supported describing the relationship between juveniles of all species combined and patch-level habitat, and indicated that juvenile birds were negatively associated with grasses and positively associated with tall shrubs (Table 2). Graminoid cover, invasives, tall shrubs, fruit abundance, and tree cover each improved one of five abundances modeled for juveniles of individual species. Habitat associations varied among species, however; juvenile Red-eyed Vireos were positively related to fruit abundance, Veeries were positively related to tree cover, and negatively associated with grass cover, and American Redstarts were positively related to tall shrubs.

The results of the analyses based on the point count subset were generally consistent with the results of the full analyses, except that they did not include fruit as an explanatory variable for adults of all species combined, or tall shrubs for adult Veeries, and included trees as predictors of adult chickadees, adult Veeries, and juveniles of all species combined (Table 3). Ovenbird adults were not analyzed with the point count subset due to insufficient data (model convergence errors).

**Table 3:** Results of likelihood ratio tests comparing top models of forest bird mist net captures with and without landscape variables and breeding abundance of forest bird in adjacent forests, using reduced dataset including only the nine sites with point count data.

	N	Top model terms	?2	DF	P
*Adults*					
ALSP	144	-**inv** +**tree** -**PC1** +**PC2** +**Bird** +year	8.4	1	0.004
REVI	55	+**fnfb** +**grm**–**inv** +**tree** -**PC1** +**year**	3.86	1	0.050
BCCH	27	-**inv** +**blsh** +**tree** +year	0.4	1	0.539
VEER	15	+fruit +**tree**	2.7	1	0.098
AMRE	14	-**grm** -**inv**	0.3	1	0.579
*Juveniles*					
ALSP	108	grm +**blsh** +**fruit** +**tree** +**PC2** +**Bird** +**year**	21.8	1	<0.001
REVI	8	+**fruit** +**Bird** -year	5.4	1	0.021
BCCH	17	-inv +**Bird**	6.1	1	0.014
VEER	34	-**grm** +**tree** -**PC1** + year	5.6	1	0.018
OVEN	8	+**PC1**	5.22	1	0.022
AMRE	7	(null)	0.9	1	0.355

Data were collected for 9 early-successional habitats during Jun.-Aug., 2006 and 2007 in Berkshire, Franklin, Hampshire, and Worcester counties, MA. Bold text indicates coefficients with 95% confidence intervals that do not include zero. Variables are as follows: ALSP = all species combined; REVI = Red-eyed Vireo; BCCH = Black-capped Chickadee; VEER = Veery; OVEN = Ovenbird; AMRE = American Redstart; Intcpt = Intercept; fnfb = ferns and forbs; gram = graminoids; lowsh = low shrubs (<2 m); inv = non-native trees and shrubs; tallsh = tall shrubs (>2 m); fruit = fruit abundance; tree = trees (>5 m); PC1 = "fragmentation"; PC2 = "early-successional"; year = year; “Bird”  =  abundance of nesting forest birds in adjacent forests.

### Bird-habitat associations – landscape-level

The top model describing the relationship between adult Red-eyed Vireos and American Redstarts and landscape-level habitat indicated that adults of these species were negatively associated with the first principal component that described increasing forest fragmentation (Table 2). Adult Veeries were negatively associated with the amount of early-successional habitat in the landscape, and the abundance of juvenile Veeries was negatively associated with fragmentation.

Top models for the abundance of adults of all species combined, juveniles of all species combined, juvenile Red-eyed Vireos, and juvenile Black-capped Chickadees all supported a strong positive relationship with bird abundance in adjacent forest during the nesting period (Table 3). Log-likelihood tests further indicated that the inclusion of landscape-level habitat significantly improved the best model for adults of all species combined and adult Red-eyed Vireos, as well as the top models for juveniles of all species combined, juvenile Veeries, and juvenile Ovenbirds.

Although generally consistent with the results of the full analyses, the analyses of the point count subset contrasted with the full analyses by indicating strong support for the negative association of all adult species with fragmentation, no association between adult American Redstarts and fragmentation, and a positive association between juvenile Ovenbirds and fragmentation (Table 3). In addition, the analyses of the point count subset did not include the amount of early-successional habitat within the landscape as an explanatory variable for adult Veeries, and did support this variable as a predictor of abundance for adults of all species combined, and juveniles of all species combined.

### Body condition

Body condition indices of American Redstart adults were negatively related to graminoid cover (β = −0.59±0.19, P = 0.008), American Redstart juveniles were positively related to PC1 (“fragmentation”; β = 0.11±0.06, P = 0.049), Ovenbird juveniles were positively related to invasives (β = 0.49±0.25, P = 0.057), and Red-eyed Vireo juveniles were positively related to tree cover (β = 0.71±0.39, P = 0.09), though this relationship was marginally significant.

## Discussion

Our study demonstrates that the inclusion of factors beyond the scale of the patch improves our ability to explain variation in the abundance of forest birds in early-successional habitats during the post-fledging period. We suggest that the inclusion of landscape characteristics into future investigations will increase the ability of investigators to understand post-fledging habitat selection by these species, and could account for the sometimes equivocal results of previous studies that based their analyses on patch-scale characteristics alone.

The relationship between captures of post-fledging forest birds in early-successional patches and the increased forest fragmentation and human land use in the surrounding landscape suggests that fragmentation reduces the abundance and/or the nesting success of adults, and hence the numbers available to utilize early-successional habitats during the post-fledging period. Abundance and nesting success are known to be negatively related to increasing forest fragmentation [14], [15], [39]–[42] and increase with forest cover [e.g. 43]. Post-hoc analyses of the abundance of the focal forest birds and fragmentation indicated the abundance of these species was not directly related to landscape metrics, suggesting it is instead the result of negative impacts of fragmentation on nesting success, and hence fewer adults with dependent young seeking early-successional habitats in which to rear their young (r = −0.29, P = 0.46). The influence of landscape we observed is consistent with Lehnen [44], who reported that in Ohio, capture rates for forest species were positively related to the amount of shrubland and mature forest in the landscape and that captures for most species were negatively related to the amount of agricultural and developed habitat in the landscape. In addition, the association between captures of forest birds during the post-fledging period and their breeding season abundance is consistent with Marshall et al. [45] and Streby and Andersen [24], who observed that post-fledging forest birds utilized clearcuts near or adjacent to the forests where they nested. The use of proximate or adjacent early-successional habitat patches by post-fledging forest birds may be influenced by additional factors, however, such as structural attributes of forest within the surrounding landscape. Recent telemetry work by Vitz and Rodewald [23] in Ohio, and Streby and Andersen [24] in Minnesota, suggest that the use of early-successional patches by post-fledging forest birds may depend on the availability of post-fledging habitat provided by dense understory vegetation within the surrounding forest (e.g. tree-fall gaps and forest wetlands). We did not account for the availability of dense vegetation in the forest understory within the landscapes surrounding our study sites, and therefore we cannot account for its effect on our observations. However, differences among site in the landscape-level availability of post-fledging habitat within forests offers a potential explanation for why the importance of landscape factors we examined were not consistent across all analyses.

In addition to landscape factors, patch-level characteristics were also important indicators of post-fledging bird abundance. The relationship we observed between captures of post-fledging forest birds and vegetation characteristics are consistent with studies that have found structurally complex vegetation to be an important component of post-fledging habitat [3], [9], [12], [13], [46]–[48]. Dense under-story vegetation may provide critical protective cover from predators for post-fledging birds, and the use of such habitat has been shown to promote their survival [3], [12], [49]. This habitat type may be of particular importance for young fledglings, which are highly vulnerable to predation due to their poor flight capability and limited mobility [2], [5], [46].

The negative relationship between captures and the cover of graminoids (grasses, sedges, and rushes) is similar to observations made in previous studies where post-fledging birds avoided or didn't use areas dominated by grasses or non-woody, herbaceous vegetation [9], [13], [50]. Although grasses and herbaceous plants may create areas of low, dense vegetation, which are characteristics generally associated with suitable post-fledging habitat [3], compared to woody vegetation, herbaceous vegetation types may lack adequate structure to provide protection from predators. Extremely dense graminoids could also inhibit foraging movement and even increase the risk of predation by concealing terrestrial predators such as snakes, which are often abundant in early-successional and grassland habitats [1], [51]. Furthermore, grass-dominated habitats tend to be structurally simple, whereas avian abundance tends to parallel structural complexity [52]–[55].

Our findings indicate that the presence of trees in early-successional habitats may attract forest birds to openings during the post-fledging season, which is consistent with other studies [56], [57], and may simply be the result of reduced contrast between forest nesting and post-fledging habitat encouraging settlement by forest-nesting birds. Alternatively, trees may provide perches that allow them to be vigilant of predators while feeding young that may still be on the ground and unable to fly. Maintaining low densities of residual trees in early-successional habitat may be beneficial for post-fledging forest birds in addition to the early-successional species that breed in these habitats [39], [58]. Many of the trees within the study sites were species of *Prunus* that were fruiting during the course of this study, and this concentrated food resource may have also contributed to the positive association between forest birds and tree cover.

In addition to vegetation structure, fruit resources are thought to be an important factor in explaining patterns of habitat use by forest birds during the post-fledging period [9], [45], [46]. However, the relative importance of fruit resources to birds compared to other factors, such as vegetation structure and invertebrate resources, likely differs among species, and potentially between juveniles and adults. Studies that have examined the habitat use of Wood Thrush and Swainson's Thrush during the post-fledging season have suggested that for juveniles, fruit is an important food resource [9], [13], [46] and a primary driver of habitat selection [13]. In contrast, other studies report little evidence for associations between forest birds during the post-fledging period and fruiting plants, or that the association of birds with fruit varies among species [51], [59]–[61]. Our findings that overall captures were positively related to fruit suggest that fruit is an important resource for birds during the post-fledging season, however our results, as well as the results of these previous studies [11], indicate that its importance as a food resource differs among bird species.

Studies of breeding birds have linked non-native invasive plants to reduced abundance and diversity of native bird species [62]-[66], greater nest predation, and decreased breeding productivity [67], [68]. To the best of our knowledge, the only other study to examine the effects of invasive plants on post-fledging birds focused on a grassland obligate species, Sprague's Pipit (*Anthus spragueii*) [69]. These authors found that juveniles reared in non-native grasslands had lower survival rates than those raised in native grasslands. While the results of our study, conducted in early-successional habitats, and the results from a study conducted in grasslands are not entirely comparable, we similarly found evidence that invasive species had a negative effect on post-fledging birds. Although the mechanism of this relationship is unclear, many invasive plant species have been linked to reduced invertebrate abundance [70]–[72], which may be a more important food resource than fruit for many species during the post-fledging season [73].

We detected few significant relationships between habitat and landscape variables and the body condition indices of forest birds. Similarly, Vitz and Rodewald [73] did not find evidence that diet influenced energetic condition for several species of forest birds during the post-fledging season. One possibility for this is that food may not have been limited in these habitats to the extent that it would have had an effect on the body condition of forest birds. For juvenile post-fledging birds, carry over effects related to natal habitat quality may be a more important determinant of condition than habitat use in the post-fledging period [73]. Further research with larger sample sizes will be required to understand the relationships between nesting habitat quality, post-fledgling habitat use and the condition of forest birds during the post-fledging season.

## Conclusions

Our finding that the inclusion of landscape and population factors beyond the scale of the patch improves our ability to explain variation in the abundance of forest birds in early-successional habitats during the post-fledging period will increase the ability of investigators to understand post-fledging habitat selection by these species, and could account for the sometimes equivocal results of previous studies that based their analyses on patch-scale characteristics alone. This study also provides further evidence that the use of early-successional habitat by forest-nesting birds during the post-fledging season is a generalized phenomenon that occurs across a wide range of species [8], [9], [13], [45], [47], [48], [59], [61], [74]. These findings suggest that management for forest birds is not necessarily in conflict with management for early-successional species, and that forest birds could benefit from management focused on providing habitat for early-successional birds, provided such management carefully weigh the potential cost of removing breeding habitat for forest-nesting species. Finally, accounting for landscape context can help target management efforts – such as in the acquisition, creation, and maintenance of openings, by identifying areas prone to edge and area effects, and colonization by invasive species [75], [76]. These results will hopefully help encourage other studies of this important, but poorly understood stage of the avian lifecycle.

## Supporting Information

Appendix S1 **Name, treatment type, location and capture rates from 15 mist-netting sites in early-successional habitats in Berkshire, Franklin, Hampshire, and Worcester counties, Massachusetts.**
(DOCX)

Appendix S2 **Mean capture rates (per 100 mist net hours), standard errors (SE) and total captures (N) from early-successional habitats during the post-fledging period of 2006 and 2007 in Berkshire, Franklin, Hampshire, and Worcester counties, Massachusetts.** Habitat associations for each species are based on Schlossberg and King (2007), DeGraaf and Yamasaki (2000), and Poole (2005). Footnotes indicate species' conservation status.
(DOCX)
