# Supplementary material for: The Effect of Local and Landscape-Level Characteristics on the Abundance of Forest Birds in Early-Successional Habitats during the Post-Fledging Season in Western Massachusetts
Source: PLoS One. 2014 Aug 29;9(8):e106398. doi: 10.1371/journal.pone.0106398 (PMC4149558; doi:10.1371/journal.pone.0106398)
Supplement: Appendix S1 — Name, treatment type, location and capture rates from 15 mist-netting sites in early-successional habitats in Berkshire, Franklin, Hampshire, and Worcester counties, Massachusetts. (DOCX) [file pone.0106398.s001.docx]

Appendix S1. Name, treatment type, location and capture rates from 15 mist-netting sites in early-successional habitats in Berkshire, Franklin, Hampshire, and Worcester counties, Massachusetts.

| Site | Treatment^a^ | Latitude | Longitude | Elevation (m) | Mean forest-bird count | Mistnet captures (per 100 hrs) |
| --- | --- | --- | --- | --- | --- | --- |
| Curtis Road | CC | 42.365225° N | 72.943098° W | 458 | NA | 128 |
| Eugene Moran | WO | 42.521240° N | 73.053879° W | 630 | NA | 45 |
| Gate 10 | CC | 42.371568° N | 72.394425° W | 330 | NA | 23 |
| Gate 8 | CC | 42.350787° N | 72.385429° W | 260 | NA | 16 |
| Savoy S.F. | CC | 42.628628° N | 73.033387° W | 581 | NA | 37 |
| Fox Den | WO | 42.389283° N | 73.002967° W | 524 | NA | 15 |
| Green River | WO | 42.645100° N | 73.237460° W | 296 | 3.67 | 23 |
| Herman Covey | WO | 42.253453° N | 72.351436° W | 175 | 3.2 | 1 |
| Leyden ‘North’ | WO | 42.682617° N | 72.603933° W | 320 | 3.87 | 5 |
| Leyden ‘South’ | WO | 42.655660° N | 72.599528° W | 170 | 3.8 | 26 |
| Muddy Brook | WO | 42.383860° N | 72.190971° W | 312 | 3 | 3 |
| Natty Pond | WO | 42.472675° N | 72.013797° W | 291 | 2.87 | 24 |
| Poland Brook | WO | 42.489644° N | 72.748425° W | 283 | 4.13 | 57 |
| Stafford Hill | WO | 42.568284° N | 73.112152° W | 507 | 5.53 | 23 |
| Taconic Trail S.P. | WO | 42.711233° N | 73.249747° W | 470 | 3.87 | 12 |

^a^ CC = clearcut, WO = wildlife opening
