# Supplementary material for: The Effect of Local and Landscape-Level Characteristics on the Abundance of Forest Birds in Early-Successional Habitats during the Post-Fledging Season in Western Massachusetts
Source: PLoS One. 2014 Aug 29;9(8):e106398. doi: 10.1371/journal.pone.0106398 (PMC4149558; doi:10.1371/journal.pone.0106398)
Supplement: Appendix S2 — Mean capture rates (per 100 mist net hours), standard errors (SE) and total captures (N) from early-successional habitats during the post-fledging period of 2006 and 2007 in Berkshire, Franklin, Hampshire, and Worcester counties, Massachusetts. Habitat associations for each species are based on Schlossberg and King (2007), DeGraaf and Yamasaki (2000), and Poole (2005). Footnotes indicate species' conservation status. (DOCX) [file pone.0106398.s002.docx]

S2. Mean capture rates (per 100 mist net hours), standard errors (SE) and total captures (N) from early-successional habitats during the post-fledging period of 2006 and 2007 in Berkshire, Franklin, Hampshire, and Worcester counties, Massachusetts. Habitat associations for each species are based on Schlossberg and King (2007), DeGraaf and Yamasaki (2000), and Poole (2005). Footnotes indicate species' conservation status.

|  |  | 2006 | | |  | 2007 | | |
| --- | --- | --- | --- | --- | --- | --- | --- | --- |
|  |  | Mean | SE | N |  | Mean | SE | N |
| ***Forest species*** |  |  |  |  |  |  |  |  |
| Yellow-bellied Sapsucker | *(Sphyrapicus varius)* | 0.14 | 0.1 | 1 |  | 0 | 0 | 0 |
| Downy Woodpecker | *(Picoides pubescens)* | 0.36 | 0.4 | 2 |  | 0.1 | 0.1 | 1 |
| Olive-sided Flycatcher^b^ | *(Contopus cooperi)* | 0 | 0 | 0 |  | 0.1 | 0.1 | 1 |
| Yellow-bellied Flycatcher | *(Empidonax flaviventris)* | 0.38 | 0.4 | 3 |  | 0.22 | 0.2 | 2 |
| Least Flycatcher^a^ | *(Empidonax minimus)* | 1.31 | 0.5 | 9 |  | 0.63 | 0.4 | 6 |
| Blue-headed Vireo | *(Vireo solitarius)* | 0.18 | 0.2 | 1 |  | 0 | 0 | 0 |
| Philadelphia Vireo | *(Vireo philadelphicus)* | 0 | 0 | 0 |  | 0.1 | 0.1 | 1 |
| Red-eyed Vireo^a^ | *(Vireo olivaceus)* | 11.7 | 7 | 71 |  | 4.69 | 3.2 | 43 |
| Black-capped Chickadee | *(Poecile atricapilla)* | 3.92 | 1.5 | 27 |  | 3.79 | 1.3 | 36 |
| Tufted Titmouse | *(Baeolophus bicolor)* | 0.18 | 0.2 | 1 |  | 0.22 | 0.2 | 2 |
| Veery | *(Catharus fuscescens)* | 4.03 | 1.3 | 29 |  | 4.58 | 2.1 | 43 |
| Swainson's Thrush | *(Catharus ustulatus)* | 0.4 | 0.3 | 3 |  | 0.1 | 0.1 | 1 |
| Hermit Thrush | *(Catharus guttatus)* | 0.38 | 0.3 | 3 |  | 0.21 | 0.1 | 2 |
| Wood Thrush^a,b^ | *(Hylocichla mustelina)* | 0.13 | 0.1 | 1 |  | 1 | 0.9 | 9 |
| Black-throated Blue Warbler | *(Setophaga caerulescens)* | 4.13 | 2.5 | 33 |  | 0.11 | 0.1 | 1 |
| Yellow-rumped Warbler | *(Setophaga coronata)* | 0.56 | 0.6 | 4 |  | 0 | 0 | 0 |
| Black-throated Green Warbler | *(Setophaga virens)* | 0.5 | 0.3 | 4 |  | 0.41 | 0.3 | 4 |
| Blackburnian Warbler | *(Setophaga fusca)* | 0 | 0 | 0 |  | 0.51 | 0.3 | 5 |
| American Redstart | *(Setophaga ruticilla)* | 4.06 | 2 | 26 |  | 2.49 | 0.7 | 24 |
| Ovenbird | *(Seiurus aurocapilla)* | 3.29 | 1.1 | 23 |  | 1.6 | 0.8 | 15 |
| Northern Waterthrush | *(Parkesia noveboracensis)* | 0.3 | 0.2 | 2 |  | 0.2 | 0.1 | 2 |
| Louisiana Waterthrush | *(Parkesia motacilla)* | 0 | 0 | 0 |  | 0.1 | 0.1 | 1 |
| Rose-breasted Grosbeak^a^ | *(Pheucticus ludovicianus)* | 0.32 | 0.2 | 2 |  | 0.72 | 0.4 | 7 |
| Scarlet Tanager^a^ | *(Piranga olivacea)* | 2.39 | 1.3 | 15 |  | 0.21 | 0.1 | 2 |
| Winter Wren | *(Troglodytes hiemalis* | 0.28 | 0.2 | 2 |  | 0.11 | 0.1 | 1 |
| Purple Finch^a^ | *(Haemorhous purpureus)* | 0.74 | 0.6 | 5 |  | 0.22 | 0.2 | 2 |
| ***Early-successional species*** |  |  |  |  |  |  |  |  |
| Black-billed Cuckoo^a,b^ | *(Coccyzus erythropthalmus)* | 0 | 0 | 0 |  | 0.11 | 0.1 | 1 |
| Ruby-throated Hummingbird | *(Archilochus colubris)* | 0.8 | 0.4 | 6 |  | 0.81 | 0.6 | 8 |
| Alder Flycatcher | *(Empidonax alnorum)* | 3.34 | 1 | 24 |  | 3.04 | 1.3 | 29 |
| House Wren^a^ | *(Troglodytes aedon)* | 0.16 | 0.2 | 1 |  | 1.89 | 0.9 | 18 |
| Gray Catbird | *(Dumetella carolinensis)* | 9.19 | 2.9 | 68 |  | 15.3 | 3.7 | 148 |
| Cedar Waxwing | *(Bombycilla cedrorum)* | 3.95 | 1.4 | 29 |  | 6.86 | 1.6 | 66 |
| Blue-winged Warbler^a^ | *(Vermivora cyanoptera)* | 0 | 0 | 0 |  | 1.02 | 0.5 | 10 |
| Tennessee Warbler | *(Oreothlypis peregrina)* | 0.38 | 0.4 | 3 |  | 0 | 0 | 0 |
| Nashville Warbler^a^ | *(Oreothlypis ruficapilla)* | 1.13 | 1 | 9 |  | 0.11 | 0.1 | 1 |
| Yellow Warbler | *(Setophaga petechia)* | 0.53 | 0.3 | 4 |  | 2.78 | 1 | 27 |
| Chestnut-sided Warbler^a^ | *(Setophaga pensylvanica)* | 6.89 | 2.1 | 48 |  | 6.11 | 2.2 | 58 |
| Magnolia Warbler | *(Setophaga magnolia)* | 2.67 | 1.2 | 20 |  | 0.31 | 0.2 | 3 |
| Prairie Warbler^a,b^ | *(Setophaga discolor)* | 0.16 | 0.2 | 1 |  | 0.63 | 0.6 | 6 |
| Black-and-white Warbler^a^ | *(Mniotilta varia)* | 1.51 | 0.6 | 10 |  | 0.8 | 0.4 | 8 |
| Mourning Warbler^c^ | *(Geothlypis philadelphia)* | 0.14 | 0.1 | 1 |  | 0.22 | 0.2 | 2 |
| Common Yellowthroat^a^ | *(Geothlypis trichas)* | 14.4 | 3.5 | 102 |  | 17.8 | 1.3 | 173 |
| Wilson's Warbler | *(Cardellina pusilla)* | 0.25 | 0.3 | 2 |  | 0 | 0 | 0 |
| Canada Warbler^a,b^ | *(Cardellina canadensis)* | 0.94 | 0.3 | 7 |  | 0.41 | 0.2 | 4 |
| Eastern Towhee^a^ | *(Pipilo erythrophthalmus)* | 0.5 | 0.3 | 4 |  | 0.2 | 0.1 | 2 |
| Field Sparrow^a^ | *(Spizella pusilla)* | 1.41 | 0.8 | 10 |  | 4.91 | 1.6 | 48 |
| Song Sparrow^a^ | *(Melospiza melodia)* | 6.67 | 3.9 | 51 |  | 17.1 | 3.4 | 167 |
| Swamp Sparrow | *(Melospiza georgiana)* | 0 | 0 | 0 |  | 0.34 | 0.3 | 3 |
| White-throated Sparrow^a^ | *(Zonotrichia albicollis)* | 1.88 | 1.3 | 14 |  | 0.2 | 0.1 | 2 |
| Dark-eyed Junco | *(Junco hyemalis)* | 0 | 0 | 0 |  | 1.1 | 1 | 11 |
| Northern Cardinal | *(Cardinalis cardinalis)* | 0.28 | 0.2 | 2 |  | 0.1 | 0.1 | 1 |
| Indigo Bunting | *(Passerina cyanea)* | 0.75 | 0.4 | 5 |  | 1.13 | 0.7 | 11 |
| Bobolink^a,b^ | *(Dolichonyx oryzivorus)* | 0.32 | 0.3 | 2 |  | 0 | 0 | 0 |
| American Goldfinch | *(Spinus tristis)* | 0.91 | 0.3 | 6 |  | 3.49 | 0.9 | 34 |
| ***Other species*** |  |  |  |  |  |  |  |  |
| Eastern Phoebe | *(Sayornis phoebe)* | 1.88 | 1.4 | 11 |  | 0.82 | 0.4 | 8 |
| Warbling Vireo | *(Vireo gilvus)* | 0.18 | 0.2 | 1 |  | 0 | 0 | 0 |
| Blue Jay^a^ | *(Cyanocitta cristata)* | 0.61 | 0.4 | 4 |  | 0 | 0 | 0 |
| American Robin | *(Turdis migratorius)* | 0.4 | 0.3 | 3 |  | 0.84 | 0.4 | 8 |
| Brown-headed Cowbird | *(Molothrus ater)* | 0 | 0 | 0 |  | 0.21 | 0.1 | 2 |
| Baltimore Oriole^a^ | *(Icterus galbula)* | 1.64 | 0.8 | 11 |  | 2.98 | 1.1 | 29 |

^a^ Significant Population Decline (P < 0.05) as determined by North American Breeding Bird Survey Results (1966-2010) for Region S30 (New England/mid-Atlantic Coast) (Sauer et al. 2012)

^b^ Partners in Flight Watch List 2012 (Partners in Flight Science Committee 2012)

^c^ Massachusetts Species of Special Concern (NHESP 2013)

**References**

DeGraaf RM, Yamasaki M (2000) New England Wildlife: Habitat, natural history, and distribution. Hanover: University Press of New England. 496 p.

Natural Heritage and Endangered Species Program (2013) Massachusetts List of Endangered, Threatened and Special Concern Species. In: Massachusetts Department of Fish and Game, editor: Natural Heritage and Endangered Species Program, Commonwealth of Massachusetts, Division of Fisheries and Wildlife.

Partners in Flight Science Committee (2012) Species Assessment Database, version 2012.

Poole AE (2005) The Birds of North America Online. Cornell Laboratory of Ornithology, Ithaca, NY.

Sauer JR, Hines JE, Fallon JE, Pardieck KL, D. J. Ziolkowski J, et al. (2012) The North American Breeding Bird Survey, Results and Analysis 1966-2011. Version 07.03.2013. USGS Patuxent Wildlife Research Center, Laurel, MD.

Schlossberg S, King DI (2007) Ecology and Management of Scrub-Shrub birds in New England: A comprehensive review. USDA Natural Resources Conservation Service, Resource Inventory and Assessment Division.
